# Folic acid usage and associated factors in the prevention of neural tube defects among pregnant women in Ethiopia: cross-sectional study

**DOI:** 10.1186/s12884-017-1506-2

**Published:** 2017-09-21

**Authors:** Meselech Ambaw Dessie, Ejigu Gebeye Zeleke, Shimelash Bitew Workie, Ayanaw Worku Berihun

**Affiliations:** 1 Department of Anatomy, School of Medicine, Collage of Medicine and Health Sciences, University of Gondar, Gondar, Ethiopia; 2 Department of Biostatic and Epidemiology, Institute of public health, Collage of Medicine and Health Sciences, University of Gondar, Gondar, Ethiopia; 3 Department of Biostatic and Epidemiology, school of public health, Wolaita Sodo University, Wolaita Sodo, Ethiopia

**Keywords:** Neural tube defects, Folic acid, Pregnancy

## Abstract

**Background:**

Neural tube defects are among the most common birth defects, contributing to miscarriage, infant mortality, severe congenital abnormalities and serious disabilities. It is burdensome to patients, caregivers, healthcare systems and society. It could be reduced if women consume a folic acid supplement before and during the early weeks of pregnancy. This study assesses folic acid usage and associated factors for the prevention of neural tube defects among pregnant women in Ethiopia.

**Methods:**

Institution based cross-sectional study was conducted on 417 systematically sampled, consented pregnant women that visited Adama hospital medical college for antenatal care during August to November 2014. Pretested interviewer administered questionnaire was used to collect socio-demographic, obstetric characteristics and folic acid usage of women.

**Result:**

About 48.4% of women took a folic acid supplement at different period of pregnancy; but, only 1.92% of women took the supplement at a protective period against neural tube defects. Age, the early timing of antenatal registration, was a preconception consulted, previous unsuccessful pregnancies and level of folic acid awareness were significantly associated with folic acid usage for prevention of neural tube defects.

**Conclusions:**

Folic acid usage during the protective period against neural tube defects among women in Ethiopia is very low, so healthcare plan to improve intake of folic acid is required.

## Background

Neural tube defects (NTDs) are congenital anomalies that result from failure of the neural tube to close between day 21 to 28 post conception [1–4]. NTDs are contributing to miscarriage, infant mortality, severe congenital abnormalities and serious disabilities and are the second most common congenital anomaly [3, 5]. NTDs account for 10% of all neonatal mortality, 10% of the burden of all congenital conditions and these defects are responsible annually for 41,000 deaths and 2.3 million disability-adjusted life years [3, 6]. NTDs are affecting 0.5–2 per 1000 pregnancies and occurring in about 2–3% live births worldwide [1, 7, 8]. Because of the lower living standard and low antenatal diagnosis and termination of pregnancy [9]; the incidence of NTDs in developing countries have been reported to be up to fourfold higher than in developed ones [10]. The lifelong medical and socioeconomic consequences of NTDs in affected children are equally known to be worse in low-resource settings [3]. A study conducted in Ethiopia reported that hydrocephalus (35.5%) and neural tube defects (27.5%) were the major leading causes of admission to hospitals for surgical procedures among children [11].

Neural tube defects have a heterogeneous and multifactorial etiology, including genetic and environmental factors as well as predisposing maternal factors [3, 8, 12]. Among known environmental factors folic acid deficiency is the most common one [12–14].

The basic characteristic of embryonic and fetal development is widespread cell division; and folic acid has a major role in DNA and RNA synthesis, for cellular development and division, the metabolism of amino acids and the regulation of homocysteine in the blood [15–18]. Folic acid deficiency is a serious problem that affects women worldwide [4]. In Ethiopia, one in every three women had a deficiency of folic acid, suggesting that these women are at a higher risk of giving birth to a baby with NTDs [19].

Folic acid intake is limited by cooking losses and poor bioavailability estimated to be from 50% to 82% [2, 19–22]. In addition, during pregnancy, maternal serum and erythrocyte concentrations of folic acid decline for several reasons: increased demand, dilution secondary to increased intravascular volume, increased folate catabolism and clearance, decreased absorption and inadequate intake [1, 6].

Many studies have documented that daily intake of 400 micrograms folic acid at least one month before pregnancy and the entire first trimester of pregnancy could reduce the risk of NTDs by up to 80% [4, 21, 23–25]. Because the neural tube closes by the fourth week after conception, it is important that folic acid be consumed on a regular basis before pregnancy [17, 26, 27].

Previous studies internationally shows different level of folic acid usage for prevention of NTDs [1, 21, 23, 25, 28–33] and discovered age [1, 21, 25, 28–30, 34], parity levels [21, 25, 29, 30], smoking status [25], history of spontaneous abortions [25], planning of pregnancy [21, 23, 28], number of ANC visits [29, 30, 35], timing of ante-natal registration [30], educational status [1, 21, 25, 30, 35], marital status [21, 25, 35], monthly income [21, 25] and number of pregnancy [28] as factors associated with preconception folic acid usage.

Although folic acid usage for prevention of NTDs were well studied internationally, there are no published reports that indicate the usage of folic acid at the recommended time among pregnant women in Ethiopia. The aim of this study was to assess folic acid usage and associated factors for the prevention of NTDs among pregnant women in Ethiopia.

## Methods

### Study settings and period

In Ethiopia pregnant women obtain antenatal care (ANC) in all government health facilities free of charge starting from one month. ANC covers a wide range of services including folic acid and iron supplement. But there is no preconception care. Based on Ethiopian mini Demographic and Health Survey 2011, 76% of urban pregnant women received ANC from a skilled provider, that is, from a doctor, nurse, or midwife. The study was conducted from August to November 2014 in Adama also known as Nazareth. Adama is a city located 99 km southeast of Addis Ababa and was a previous capital of the Oromia region from August to November 2014.

### Study design and population

An institution based cross-sectional study was conducted on pregnant women who visited Adama hospital medical college, the former Haile Mariam Mamo memorial hospital, for the ANC. Women in 2nd and 3rd trimester of pregnancy were included in the study. Women who come repeatedly during the data collection time were excluded.

### Sample size determination and sampling procedure

The sample size was determined using a formula for a single population proportion and calculated by Open Epi Info version 3.5.3 statistical software package based on the following assumptions: the target population greater than 10, 000, since the proportion of folic acid usage for NTD prevention in Ethiopia was unknown 50% was used, significant level at 95% confidence interval and margin of tolerable sampling error 5%. After calculating sample size was 383. After adding 10% for non-response the final sample size becomes 422. The study participants were selected by using a systematic random sampling method. After taking informed consent, the participants were interviewed in the waiting room before entering to ANC unit.

### Data collection tools and study variables

**Fig. 1:**
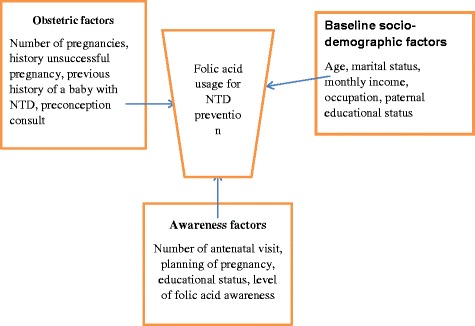
Conceptual framework to assess folic acid usage and associated factors for prevention of NTDs among pregnant women developed from literatures

A pretested interviewer administered questionnaire written in English was prepared. The questionnaire was translated into the local language (Amharic) to the guardians then translated back to English to evaluate its consistency. A detailed history of socio demographic characteristics (age, marital status, educational status, monthly income which was scaled as birr per month, occupation, husband educational status; obstetric characteristics (number of pregnancies, preconception consult which means asking advise from healthcare provider by own initiation, number of antenatal visit, planning of pregnancy, history of unsuccessful pregnancy, previous history of a baby with NTDs) and awareness and use of folic acid were assessed by interviewing the women. The dependent variable was folic acid usage for NTD prevention. The independent variables were socio demographic characteristics, obstetric characteristics and level of information about folic acid. Figure 1 shows the conceptual framework for folic acid usage and associated factors. Folic acid user for NTD prevention was defined in this study as a woman who took folic acid at least one month before pregnancy and continue for three months after pregnancy. Total user of folic acid were women who took folic acid at any time during current pregnancy for at least one month.

### Data processing and analysis

Data were entered into Epi-Info version 3.5.3 and exported to SPSS version 20 statistical software for analysis. Descriptive statistics were carried out using percentage and presented in tables. Bivariate logistic regression analysis, Chi square and Fisher’s exact test were conducted to assess the association between dependent and independent variables. Odds ratio with 95% confidence interval and *p*-value were used to evaluate the association between the dependent and independent variables. A *p*-value less than 0.05 was considered as statistically significant.

### Ethical considerations

The study was conducted following ethical approval by the University of Gondar, College of Medicine and Health Sciences, School of Medicine Ethical Committee. Permission was taken from the hospital administrators. Informed consent was taken from the study participants. Participation in the study was voluntary and refusal was possible. The study did not involve any harmful procedure. To keep confidentiality codes were used and an unauthorized person did not have access to the data.

## Results

A total of 422 pregnant women attending ANC in Adama hospital medical college from August to November 2014 were approached to fill a questioner but 417 women willing to fill it. The response rate was 98.8%. The mean respondent age was 25.3 years, 45.3% of the respondents were aged less than 25 years, 49.4% of the women were aged between 25 and 35 years, and 5.3% were aged 35 and above. The minimum age was 16 and the maximum was 41. Household monthly income was less than 2500ETB in 57.3% of the respondents and greater than or equal to 2500 to 42.7% of the respondents. Table 1 shows the detail socio demographic characteristics of the women.

**Table 1:** Socio demographic characteristic of women attending ANC in Adama Hospital Medical College 2014 *N* = 417

Variables		Frequency	Percent
Age	<25 years	189	45.3
	25–34 years	206	49.4
	= > 35 years	22	5.3
Marital status	Not married	20	4.8
	Married	337	95.2
Educational status	Illiterate	38	9.1
	Primary education	156	37.4
	Secondary education	146	35.0
	College diploma and degrees	77	18.5
Monthly income	<2500 ETB	239	57.3
	>2500 ETB	178	42.7
Occupation	Housewife	242	58
	Working women	175	42
Paternal educational status	Illiterate	26	6.2
	Primary education	110	26.4
	Secondary education	151	36.2
	College diploma, degree	130	31.2

About 46.3% the women were primigravidae while 49.2% had 2–4 pregnancies and 4.6% had 5 or more pregnancies. The number of pregnancies ranges from 1 to 10 (2 ± 1.3). The mean gestational age at the time of interview was 29.2 weeks with the minimum 13 week and maximum 40. About 11.3% of the women consulted a healthcare provider when they plan to become pregnant. Among those who consulted healthcare providers 93.6% could mention what was done by a healthcare provider when they consult them. Advice was reported by 4.5%, general check up by 72.7% and general checkup plus folic acid supplement by 18.2%.

Of al1 9.7% of women comes for the first visit while 63.3% comes for the 2nd to 4th visit and 17.0% comes for 5th and more visits at the time of interview. The month of first ANC visit ranges from one month to nine months (mean = 3.8, median = 4, mode = 4). 42.2% of the women made their first ANC visit in the first trimester, 53.5% in the second trimester and 4.3% in the third trimester of pregnancy. 19.4% had a history of unsuccessful pregnancy in a lifetime and 1.7% of the women had a history of a baby with other birth defects.

The current pregnancy was planned by 71% of the women. Among women who planned pregnancy 48% stopped using contraceptive without consulting healthcare provider, 21% consulted care giver before stopping birth control and 31.1% did not use birth control. Table 2 shows the detail obstetric history of the women.

**Table 2:** Obstetric history of women attending ANC in Adama hospital medical college 2014 *N* = 417

Variable	Frequency(n)		Percent
Number of pregnancy	Primigravida	193	46.3
	2–4	205	49.2
	> = 5	19	4.6
Preconception consult	Yes	47	11.3
	No	370	88.3
What has been done during consultation (*N* = 44)	Advice	2	4.5
	General check up	32	72.7
	General checkup & folic acid supplement	8	18
	Remove birth control	2	4.5
Month of first ANC Visit	First trimester	176	42.2
	Second trimester	223	53.5
	Third trimester	18	4.3
Number of ANC visit	first visit	82	19.7
	2nd -4th	264	63.3
	More than 4th	71	17.0
History of unsuccessful pregnancy in life time	No	345	80.6
	Yes	81	19.4
Type of unsuccessful pregnancy in life time	Spontaneous abortion	63	15.1
	NTD	3	0.7
	Other(still birth, congenital anomaly)	15	3.6
History of a baby with birth defect	No	410	98.3
	Yes	7	1.7
Diagnosed Diabetes mellitus	No	409	98.08
	Yes	8	1.92
Planning of pregnancy	Yes	296	71
	No	121	29
Action when plans to become pregnant (*N* = 296)	I stopped without consulting healthcare provider	142	48
	I consulted healthcare provider before stopping BC	62	21
	I did not use birth control	92	31.1

Folic acid was prescribed for 54.9% of the women for the current pregnancy. Of those women, 77.3% took folic acid daily, 10.9% forgot 1–2 times per week, 6.6% interrupted, and 5.2% never took the supplement. Among 217 women who took folic acid during current pregnancy 6.9% used less than one month, 182 (83.9%) used for one to three months, 15 (6.9%) used for four to six months and 5 (2.3%) for seven months and above. Among the prescribed women, 3.5% started to take folic acid supplements before pregnancy and 11.6% started in the first trimester. The remaining 51.1% and 33% of the women started taking the supplement at their second and third trimester of pregnancy respectively. The overall users of folic acid supplement become 48.4% (202/417). But only 8 of the users took folic acid at a protective period against NTD. This makes the total user of folic acid supplement for NTD prevention 1.92% (Table 3).

**Table 3:** Folic acid usage and associated factors for NTD prevention among women attending ANC in Adama Hospital Medical College 2014

Variable	Category	Frequency	Percent
Prescribed for folic acid supplement for current pregnancy (*N* = 417)	Yes	229	54.9
	No	188	45.1
Do you take as recommended(*N* = 229)	yes I took as recommended	177	77.3
	sometimes I forgot	25	10.9
	Interrupted	15	6.6
	I never took FA	12	5.2
When do you start to take folic acid (*N* = 229)	before I become pregnant	8	3.5
	first trimester	26	11.5
	Second trimester	118	51.1
	Third trimester	77	33.9
Total month of folic acid intake(*N* = 217)	Less than 1 month	15	6.9
	1–3 month	181	83.9
	4–6 month	16	6.9
	7 month and above	5	2.3
Level of folic acid awareness	Uninformed	328	78.7
	Somewhat informed	39	9.4
	Informed	50	12
Total user of folic acid	Yes	202	48.7
	No	214	51.3
Folic acid user for NTD prevention	Yes	8	1.9
	No	409	98.1

Folic acid usage for NTD prevention was significantly associated with advanced age (*p* value =0.02 and OR = 9.4 with 95% CI 1.14–76.8), early antenatal registration (*p* value =0.03 and OR = 0.974 with 95% CI 0.96–0.99), number of ANC visit (*p* value = 0.04), consultation during preconception period (*p* value = 0.000 and OR = 0.063 with 95% CI 0.15 to 0.275), previous history of unsuccessful pregnancies (*p* value = 0.04 and OR = 4.3 with 95% CI 1.1 to 17) and level of folic acid awareness (*p* value = 0.00).

There was no significant correlation between folic acid usage and the women’s educational status, occupation, monthly household income, number of pregnancy and partner’s educational status.

The variables planning of pregnancy, having a history of a baby with a birth defects and experiencing illness during current pregnancy did not reach a significant level. But all of these had correlation with folic acid usage for NTD prevention. Table 4 shows the detail of folic acid usage and associated factors for NTDs prevention.

**Table 4:** Statical Analysis of Folic Acid Usage and Its Dependent Variables

Covariates		Folic acid user		OR(95% CI)	*P*-value
		No	Yes		
Number of pregnancy	Primigravida	190(98.4%)	3(1.6%)	1.45(0.341–6.13)	0.426
	Other	219(97.8%)	5 (2.2%)		
Preconception consult	Yes	39(83%)	8(17%)	0.83(0.729–0.944)	0.000
	No	370(100%)	0(0.0%)		
Have ANC visit for current pregnancy	Yes	327(97.6%)	8(2.4%)	0.98(0.96–0.993)	0.171
	No it is first	82(100%)	0(0.0%)		
Month of first visit of ANC	First trimester	305(97.4%)	8(2.6%))	0.97(0.96–0.99)	0.032
	Other	103(100%)	0(0.0%)		
Number of ANC visits	1st visit	82(100%)	0(0.0%)	Not applicable	.046
	2nd to 4th visit	260(98.5%)	4(1.5%)		
	>4th visit	67(94.4%)	4(5.6%)		
History of unsuccessful pregnancy in life time	No	332(98.8%)	4(1.2%)	4.31(1.056–17.62)	0.04
	Yes	77(95.1%)	4(4.9%)		
History of a baby with birth defect	No	403(98.3%)	7(1.7%)	9.6(1.016–17.62)	0.128
	Yes	6(85.7%)	1(14.3%)		
Planning of pregnancy	Planned	288(97.3%)	8(2.7%)	0.97(0.95–0.992)	0.063
	Unplanned	121	0(0.0%)		
Level of folic acid awareness	Uninformed	328(100%)	0(0.0%)	Not applicable	0.000
	Somewhat informed	39(100%)	0(0.0%)		
	Informed	42(84%)	8(16%)		

## Discussions

Intake of folic acid at least one month before pregnancy and the entire first trimester is highly recommended to decrease birth defects, particularly NTDs [10, 17, 36]. But, the prevalence of folic acid usage at the recommended time in Adama, Ethiopia was found to be 1.92%. Ethiopia is one of the developing countries where the incidence of NTDs is highly prevalent and where 1/3 of women were affected by folate deficiency [10, 19].

In the present study, pregnancy was planned by 71% of the interviewed women, which was consistent with the planning of pregnancy in Croatia [21]. It was higher than usual reports of 50% of pregnancies, being unplanned [7, 16, 34].

Of those women who planned pregnancy 69% used contraceptive and 30.4% inform the healthcare provider before ceasing the contraceptive. As compared to survey in Europe, where 18% of the women were consulted healthcare provider prior to stopping contraceptive, the finding in this study was higher [24]. About 11.3% of the women consulted healthcare providers, particularly about their pregnancy when they plan to become pregnant. Despite all these convenient conditions to give a folic acid supplement only 2.7% of the planning women were prescribed for folic acid during the protective period against NTD, which was very low as compared with 21% usage in Croatia that had similar levels of planned pregnancy [21].

Even though the number of women who consulted healthcare providers prior to stopping contraceptive in the present study was higher than surveyed women in Europe, usage in Ethiopian women was extremely low as compared to usage by 28% plan to become pregnant and 55% pregnant women in Europe [24].

In this study, 1.92% of the women were diabetic, 0.7% women had previous history of NTD pregnancies, both of which are high risk factors for occurrence and recurrence of NTD [3]. However, none of them were user of folic acid at the recommended time. This finding was in contrast to the study done in Nigeria, where 40% of women with a previous history of NTD pregnancy were the user at protective period [1].This difference may be because of the difference in health policy, i.e. there is preconception care in Nigeria but not in Ethiopia [37].

The occurrence of NTDs among spontaneously aborted fetuses is 10-fold higher than the rate of NTDs at birth [17]. In this study, 15.1% of the study subject had at least one spontaneous abortion in their lifetime. So those women are at high risk for having a baby affected by NTDs.

Women whose age were above 25 were 9.4 fold user of folic acid at protective period than women age 25 and below which was consistent with other studies in USA [27, 34], Croatia [21], Lebanon [25], and Tanzania [29]. This finding was in contrast to the other study in Lebanese where young women were more user [28].

Early timing of antenatal registration and number of ANC visits also positively associated with folic acid usage for NTD prevention as other studies done in Tanzania, India, Honduras [29, 30, 35].The study findings were consistent with other studies who have reported that the level of folic acid awareness predicts folic acid usage at protective period.

In this study, women who consulted healthcare providers when they plan to become pregnant were 6.3 times more likely to take folic acid at protective period than who did not consult. Similar to studies in Lebanon and Nigeria previous history of unsuccessful pregnancies was positively associated with intake of folic acid at protective period against NTDs [1, 25].

Data reported during the last 7 years from USA, Australia, Lebanon, Iran, United Arab Emirate and Nigeria shows folic acid usage at protective period against an NTDs range from 2.5% to 76% [1, 5, 10, 15, 25, 31, 36, 38]. The prevalence of folic acid users in this study is even lower than the least range. However, it is higher than a study done among Honduran women in 2007 which was 0.2% [10].

In Ethiopia, there are health facilities distributed in every city and villages in which healthcare providers deliver different medical and health need of the society including the ANC. However, folic acid usage is very low.

The poor intake of folic acid among Ethiopian women could be a result of low recommendation from policy makers, less prescription, and recommendations by healthcare providers and lack of awareness about folic acid supplements, its importance and the recommended time among women. The other one may be failures of healthcare providers to prescribe folic acid at protective time and lack of preconception care.

The study is a hospital based study on women who seek routine ANC. The result may only reflect usage among women who might have high health service seeking behavior. Another limitation of the study was it is conducted in one region, relatively a big city. So the finding of this study may not be generalized to other regions of the country, especially rural areas and small towns. Despite those limitations, the present study provides some insight into the practice of women for usage of folic acid for prevention of neural tube defects.

## Conclusions

Even though preconception folic acid intake is simple measures to prevent many severe birth defects, particularly NTDs; in Ethiopia it is unnoticed by policy makers, less prescribed at the protective period by healthcare providers, underused by pregnant women and/or women planning pregnancy.

The folic acid intake rate for NTD prevention in the present study indicates special intervention plans should be developed by health policy makers. This intervention could be screening childbearing-age women for folic acid use and providing information on the benefits of folic acid supplementation, as well as training healthcare providers on NTD and other birth defect prevention mechanisms, providing education for the whole community even early in high school level using media and other methods.

The other most important intervention that could be done by policy makers and other stakeholders is starting preconception care for women who plan to become pregnant. This action might improve folic acid usage for preventing NTDs and other birth defect and also early ANC registration.

Folic acid rate of intake in this study also indicates urgent action is required from healthcare providers to improve usage at protective period against NTDs and other congenital anomalies. So further effort is required and it is necessary to have health education on NTDs and folic acid usage during premarital health examination, when providing family planning services and during other contact with the community.

## Abbreviations

ANC: Ante natal care
CI: Confidence interval
ETB: Ethiopian birr
NTD: Neural tube defect
